# A Novel Three-Dimension Printed Individualized Curved-Needle-Based Interstitial Brachytherapy for Re-Irradiation in Uterine Fundus-Involving Recurrent Cervical Cancer: A Case Report

**DOI:** 10.3390/curroncol33020108

**Published:** 2026-02-12

**Authors:** Yangyi Zhang, Jie Zhang, Meiling Zhong, Lang Yu, Chunli Luo, Junfang Yan, Ke Hu

**Affiliations:** 1 Department of Radiation Oncology, Peking Union Medical College Hospital, Chinese Academy of Medical Science & Peking Union Medical College, Beijing 100010, China; zhangyangyi@pumch.cn (Y.Z.); zhangjie58@pumch.cn (J.Z.); yulang@pumch.cn (L.Y.); lchunli4887@163.com (C.L.); 2 Department of Oncology, Jiangxi Maternal and Child Health Hospital, Nanchang 330006, China; jxyxy@ncu.edu.cn

**Keywords:** curved-needle interstitial brachytherapy, re-irradiation, recurrent cervical cancer

## Abstract

Background: Re-irradiation in recurrent cervical cancer presents significant therapeutic challenges, particularly in those with a short interval since prior treatment. Brachytherapy is an ideal re-irradiation method, but regular intracavitary brachytherapy combined with interstitial brachytherapy (ISBT) is restricted by some challenging tumor anatomical locations, which can be optimized through a three-dimensional (3D)-printed curved-needle ISBT system. Case Presentation: A 41-year-old woman diagnosed with cervical mucinous adenocarcinoma developed central pelvic relapse, with tumor extension to the uterine fundus, within two years after completing standard concurrent chemoradiotherapy (CCRT). The patient subsequently received 3D-printed individualized curved-needle-based, MRI-guided adaptive ISBT, in combination with external beam radiotherapy (EBRT) and chemotherapy. This comprehensive treatment approach achieved 45 months of overall survival (OS), 37 months of local control (LC) and progression-free survival (PFS) with grade 3 proctitis and grade 3 cystitis and no fistulation or perforation. Conclusions: The 3D-printed individualized curved-needle ISBT is a re-irradiation option that provides satisfactory LC and prolonged survival with acceptable adverse effects for recurrent tumors located in the distal uterine fundus in cervical cancer. This technique is particularly valuable when intracavitary and traditional interstitial applicators are unsuitable due to the unique location of the tumor.

## Simple Summary

Local treatment for recurrent cervical cancer is a significant challenge. Brachytherapy is an excellent choice for re-irradiation in those case, because of its rapid dose fall-off, preventing severe treatment side effects. Intracavitary brachytherapy combined with interstitial brachytherapy can deliver high conformal dosage to irregularly shaped tumors. However, the traditional straight-needle-based interstitial brachytherapy is not able to reach to some unique relapse locations. In this case, we report on a cervical cancer patient with a recurrent tumor in uterus fundus, treated by a novel curved-needle interstitial brachytherapy, who reached a 37-month local control and 45-month overall survival with acceptable side effects.

## 1. Introduction

Cervical cancer is the tumor with the fourth highest mortality and morbidity in females worldwide [1]. Radiotherapy, combined with concurrent chemotherapy, is the main treatment strategy for local advanced-stage cervical cancer [2]. Pelvic relapse remains a pattern of treatment failure, up to 30~45% in recurrent cases, in cervical cancer cases that received chemoradiotherapy (CCRT) as initial treatment and is associated with a poor prognosis [3,4]. For those central relapse cases with prior radiotherapy, pelvic exenteration with intraoperative radiotherapy, radical hysterectomy in selected patients or individualized radiotherapy combined with chemotherapy are recommended in the NCCN guideline [5]. High rates of severe adverse events, including morbidity and perioperative mortality, are reported in cases of extensive salvage surgery, with significantly reduced quality of life [6,7]. Re-irradiation as another salvage therapy option remains a significant clinical challenge due to the high incidence of toxicity, including proctitis and cystitis, particularly for in-field relapses occurring within a short time interval [8,9]. Brachytherapy enables high-dose radiation delivery to the tumor while sparing organs at risk (OARs) due to fast dose fall-off in recurrent cervical cancer [8,10]. Interstitial brachytherapy (ISBT) further improves dose distribution to large and asymmetrical tumors or challenging anatomy location [11,12,13]. Our team previously developed a novel 3D-printed, individualized curved-needle ISBT system, which offers flexible needle insertion angles via an intracavity template [14]. In this case, we report the first follow-up data from a patient treated by the curved-needle based ISBT with a uterine fundus recurrence.

## 2. Case Presentation

### 2.1. Initial Treatment (2019–2020)

A 41-year-old woman with no significant family history was diagnosed with stage IIIB cervical HPV-related mucinous adenocarcinoma (Federation of Gynecology and Obstetrics (FIGO) Staging System, version 2018) with elevated cancer antigen 125 (CA125) to 57.2 U/mL in July 2015. Whole-pelvis radiotherapy was performed from December 2019 to January 2020. The clinical target volume (CTV) included the uterus, cervix, vagina, parametria, and pelvic lymph node drainage areas. A total dose of 45 Gy in 25 fractions was delivered using intensity-modulated radiotherapy (IMRT), with sequential boosts to the parametrial tissue and pelvic lymph nodes achieving total doses of 53 Gy and 60 Gy, respectively. Six fractions of 2D brachytherapy were administered sequentially with an equivalent dose of 2 Gy (EQD2) equal to 40 Gy (α/β = 10) at point A. Concurrent and sequential chemotherapy with six cycles of nedaplatin and paclitaxel liposome were applied from September 2019 to February 2020.

Post-treatment PET-CT in February 2020 demonstrated a mild metabolic response, indicating possible residual tumor cells. Follow-up CT scans and serum tumor markers, including CA125, showed no abnormal findings over the subsequent two years.

### 2.2. Disease Recurrence (2022)

**Figure 1:**
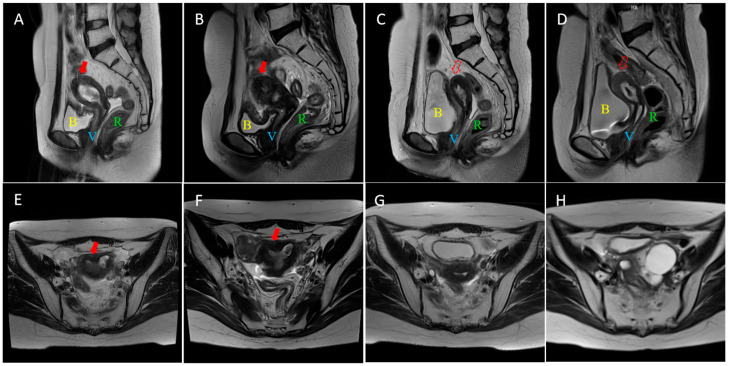
Pelvic MR images after recurrence. Representative sagittal and cross-sectional MR images of uteri fundus at recurrence (July 2022, (**A**,**E**)), after salvage EBRT/before ISBT (September 2022, (**B**,**F**)), 3 months after salvage RT (February 2023, (**C**,**G**)) and 37 months after salvage RT (May 2025, (**D**,**H**)). The red arrow indicates the recurrent cancer in the uterus fundus, and the red dash-line arrow indicates the recovered uterus fundus. B in yellow color: Bladder, V: Vagina, R: Rectum.

In February 2022, 24 months after initial treatment, the patient developed irregular vaginal bleeding. CA125 elevated to 90.6 U/mL. PET/CT in March 2022 revealed thickening of the uterine cervix and body, indicating local control failure without pelvis lymph node involvement. Biopsy of the uterine cervix confirmed pathological evidence of tumor recurrence. Physical examination revealed bilateral parametrial extension reaching the pelvic sidewall on the right and MRI demonstrated extensive involvement of the uterine cervix and body, with distal extension to the uterine fundus, (Figure 1A,E).

### 2.3. Salvage Treatment (2022)

From 4 August to 21 October 2022, the patient received EBRT and brachytherapy. The CTV for IMRT encompassed the uterine cervix, uterine body, vagina, and parametria. A prescription dose of 40 Gy in 20 fractions was delivered to 95% of the planning target volume (PTV).

**Figure 2:**
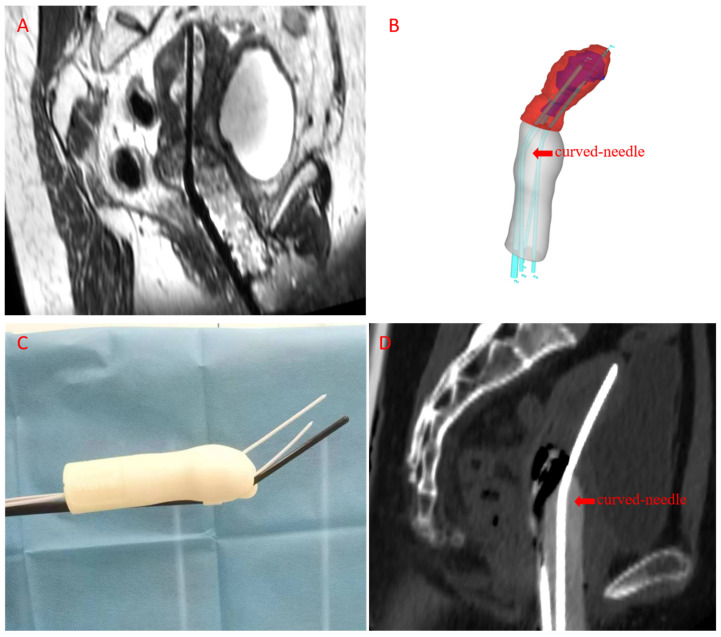
Illustration of 3D-printed curved-needle template production. (**A**) MR images for acquisition of vaginal structural data; (**B**) 3D reconstruction of template and designation of needle paths; (**C**) 3D-printed individualized template; (**D**) curved-needle path under CT image.

**Figure 3:**
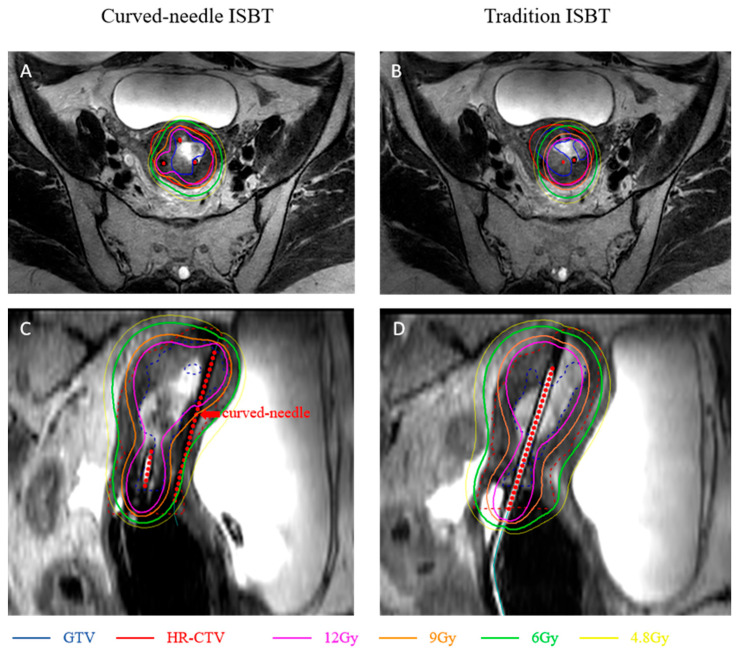
Dose distribution in traditional ISBT and curved-needle ISBT. (**B**,**D**) show dose distribution in traditional ISBT, (**A**,**C**) show dose distribution in curved-needle ISBT. Needles or intracavity tube are shown with a red dotted line. Colored full line illustrates different dose gradient lines from 200% prescription dose (12 Gy) to 80% prescription dose (4.8 Gy). HR-CTV and GTV delineation are illustrated in red and blue full lines in (**A**,**B**) and dashed line in (**C**,**D**).

For brachytherapy, individualized curved-needle based ISBT was chosen, because a commercial intracavitary applicator or straight-needle ISBT with individual template were not able to deliver a high enough dose for the extensive tumor shown in the Table 1. A CT image for template design was acquired with the patient’s vagina packed with meglumine diatrizoate gauze piece. An individualized template was generated through structural data reconstructed from a CT image by Mimics 21 (version 21.0; Materialize, Leuven, Belgium) with a curved interstitial needle arrangement designed by Geomagic Design Direct 2014 software (3D Systems), printed by a 3D-printer (Lite 600, Luen Thai, Shanghai, China), shown in Figure 2. Curved needle paths were designed within 1.4 cm of the exit template and the curve angle was 24 degrees, shown in Figure 2D. A prescribed dose of 30 Gy in five fractions (EQD2 equal to 40 Gy, alpha/beta = 10) was planned to be delivered to the uterine cervix, uterine body, and parametrial. The final treatment plan with a curved-needle-based individual template showed ideal dosage delivery in both GTV and HR-CTV, while insufficient in a straight-needle-based individual template with similar OARs dosage, shown in Figure 3 and Table 1. Acute Grade 2 diarrhea and grade 2 neutropenia, based on Common Terminology Criteria for Adverse Events (CTCAE) version 5.0, occurred during radiotherapy.

Paclitaxel and platinum (TP) chemotherapy was administered with six cycles. Three cycles of bevacizumab concurrent with chemotherapy were administered after radiotherapy and 3 cycles after chemotherapy were administered. Bevacizumab was discontinued due to gross hematuria in January 2023.

### 2.4. Follow-Up (2022–2025)

#### 2.4.1. Outcome

Serial MRI scans (Figure 1) demonstrated continuous shrinkage of the recurrent mass. Both abnormal tumor signal intensity and uterine volume decreased when comparing MR images obtained three months after salvage radiotherapy (Figure 1C,G) with those at the time of initial recurrence (Figure 1A,E), with mild residual tumor signal. Subsequent follow-up MR images revealed continued shrinkage of the uterus, and no abnormal tumor signals were detected on the most recent scans (Figure 1D,H). The patient remains under routine surveillance. The blood test showed CA125 decreased from 90.6 U/mL to below the normal range within the first month after salvage treatment and remained stable throughout the follow-up period. Overall survival (OS) after recurrence reached 45 months in November 2025. Local control (LC) and progression-free survival (PFS) were confirmed by chest and abdominal CT and pelvis MR scan, and reached 37 months in March 2025.

#### 2.4.2. Toxicity

Late radiotherapy-associated adverse events included grade 3 cystitis with gross hematuria since January 2023 (3 months after radiotherapy) and grade 3 colitis with hematochezia and erosion in descending colon since May 2023 (10 months after radiotherapy). All adverse events were alleviated after symptomatic treatment. No fistula, perforation or other adverse events were observed. The adverse event grades are based on CTCAE version 5.0.

## 3. Discussion

Cervical cancer is a leading malignancy among women worldwide. Adenocarcinoma is a less common, heterogeneous subtype of cervical cancer, with lower response rates to chemotherapy and radiotherapy, poorer prognosis, and higher recurrence risk compared to squamous cell carcinoma, requiring more intense treatment [15,16,17]. Radiotherapy is a cornerstone of treatment in cervical cancer, particularly for locally advanced disease [10]. Despite this, over one-third of patients experience treatment failure, with 80% of recurrences occurring within two years [3,4]. Pelvic relapse constitutes a significant failure pattern (48.7%), with central sites (vaginal vault, cervix, uterus) being commonly involved [3]. Local control for pelvic recurrent tumor was achieved by salvage surgery or radiation. Pelvic exenteration is the most common salvage surgical option, in which 50–60% 5-year OS was reported, along with 32–84% post-operative complications and ~10% mortality. In addition, this surgery showed serious negative effects on the quality of life among patients due to fistulization and loss of partial functions of the gastrointestinal and urogenital systems [6,7]. Radical hysterectomy is an alternative salvage surgical option for selected patients whose tumor is confined to cervix and tumor size is less than 2 cm, with lower complications and morbidity, however with shortened overall survival in long-term follow-up (28 months versus 14 months) [7]. Radiotherapy for those who have not been exposed to EBRT is an ideal treatment option; however, the majority of recurrent cervical cancer patients have received whole pelvis radiation or more extensive field radiation in their initial treatment. In this case, the recurrent tumor had reached the pelvic wall and salvage surgery may not have been able to clear all relapsed tumors, and this patient chose radiotherapy to preserve the bladder and rectum functions for a better quality of life.

Re-irradiation is a challenging issue in radiotherapy, especially with a short interval since prior treatment. While stereotactic body radiotherapy (SBRT) offers an alternative for small volumes, usually applied in isolated lymph nodes or inoperable pelvic wall metastasis, brachytherapy remains as the ideal model for re-irradiation, especially in central relapse, due to its rapid dose fall-off, thus improving survival and local control while reducing toxicity. For large or asymmetrical recurrent tumors, interstitial needles are critical for optimal dose shaping [14]. Reported EQD2 doses in high-dose-rate brachytherapy during re-irradiation range from 40 to over 100 Gy in 4–6 fractions, with 2-year LC rate of 15–52% and 2-year OS 52–78%. Grade 3–4 toxicity rates (e.g., fistulae, ulceration, bleeding) are reported in 33–51% of patients, including colitis, cystitis and fistula before 2020, reviewed by Shen et al. [8]. The 3D-printed individual template and MR-guide technique enhanced accuracy of dose delivery and improved the outcomes and decreased G3–4 adverse events morbidity of high-dose-rate ISBT, see Table 2 [18,19,20,21,22]. Our team firstly reported efficacy and safety data of 3D-printed vaginal applicator ISBT for central pelvic-recurrent cervical cancer [23]. One in nine of our reported re-irradiated cases developed rectovaginal fistula. Except for intracavity ISBT, several templates with different entry paths were studied. Zhang et al. showed a case of trans-inguinal ISBT treated in an inguinal lymphatic recurrent cervical cancer patient with 36-month progression free survival and minor skin adverse events [24]. Perineal-based ISBT were also reported to achieve over 80% LC rate in over 35 months, followed up with 8–20% long-term G3–4 grade gastrointestinal or urogenital adverse events [25,26]. Other percutaneous ISBTs through abdominal walls were also applied in pelvic or abdominal oligometastatic gynecologic cancer patients [27].The 3D-printed curved-needle system represents a significant advance, enabling treatment of distal tumors via an intracavitary approach without percutaneous penetration, thereby reducing patient discomfort and intra-abdominal complications, shortening needle path and increasing accuracy. In this case, ISBT with a straight needle through an intracavity template or perineal-based template were not able to meet the clinical requirements for recurrent fundus involvement, as shown in Figure 1. More cases with curved-needle ISBT in anatomically challenging recurrent tumors should be followed up to prove its superiority in selected cases and explore which type of recurrent patterns are required for curved-needle ISBT.

## 4. Conclusions

This case demonstrates the successful use of a novel curved-needle interstitial brachytherapy technique combined with EBRT and systemic therapy to achieve prolonged OS (over 45 months) and LC (over 37 months) with durable adverse events, in a patient with a challenging uterine fundus recurrence of cervical adenocarcinoma. This approach offers a promising, less invasive salvage option for distally located recurrences that are inaccessible with conventional brachytherapy methods.

## Figures and Tables

**Table 1:** Dosimetric comparison (one fraction) between traditional ISBT and curved-needle ISBT.

Dosimetric Parameters	Traditional ISBT	Curved-Needle ISBT
HR-CTV D90 (cGy)	532	629
GTV D98 (cGy)	500	798
Bladder D2cc (cGy)	544	554
Rectum D2cc (cGy)	184	193
Sigmoid D2cc (cGy)	419	429
Bowel D2cc (cGy)	98	99

**Table 2:** Reviews on HDR-ISBT in re-irradiation from 2020 to 2025 [18,19,20,21,22].

Study	Sample Size	Recurrent Site	Treatment Regimen	Delivered Dose (EQD2 and Gy/fx) Assumingα/β = 10	Imaging/Template	Local Control	Other Outcomes	Late Toxicities	Prognostic Factors	OARs D2cc (EQD2 a/b = 3)
Zhang et al.	1	Uterus Fundus	Curved-needle based ISBT + EBRT	ISBT: 6 Gy × 5 fractions; EBRT: 40 Gy in 20 fractions, cumulative EQD2 equals to 80 Gy	MR-guided	LC: 37 month (not reached)	OS: 45 month (not reached)	Grade 3 cystitis and Grade 3 colitis	NA	Rectum: 53 Gy * Bladder: 79 Gy * Sigmoid: 66 Gy * Small Intestine: 58 Gy *
Wang 2024 (J Gynecol Oncol) [20]	45	Pelvic recurrences or distal recurrences	CT-guided HDR-ISBT (3D-PNCT) + EBRT	EBRT: Not Mentioned ISBT: 4–7 Gy × 3–8 fractions; EQD2 ~48–70 Gy (calc)	2-year LPFS: 30.0%; 5-year LPF: 25.7% TRR: 66.7%	CT-guided; 3D-PNCT	2-year OS: 49.5% 2-year OS: 34.0% Mean OS: 23.2 months	G3–G4 toxicities: 20.0% Proctitis 4.4% Cystitis 4.4% Dermatitis 2.2% Mucositis 2.2%	Squamous cell carcinoma; HR-CTV D90 ≥ 45 Gy; tumor size; pelvic recurrence type; recurrence interval	Not Mentioned
Guo 2023 (Pak J Med Sci) [18]	36	Central and non-central pelvic recurrences	HDR-ISBT + EBRT	EBRT: 27–36 Gy Not Mentioned	CT-guided	1-year LC: 94.0% 2-year LC: 90.6%	1-year OS: 88.5% 2-year OS: 76.4%	≥Grade 2 toxicity: Bladder: 6/36 Rectum: 4/36	Maximum tumor diameter	Not Mentioned
Engineer 2022 (Clinical Oncology) [19]	90	Vaginal apex	CT-guided HDR-ISBT + EBRT	EBRT: 46–50 Gy ISBT: 20 Gy × 5 fractions; EQD2 23 Gy (calc) D90mean: 72 Gy (46–82 Gy)	CT-guided; cylinder + needles	5-year LC: 87.6%	G1–G3 GI toxicity: 22%; G1: 13.3%, G2: 5.6% G3: 3.3%, One case of fistula; G1–G3 GU toxicity: 18%	5-year OS: 70.7%	Parametrial extent; nodal involvement	Rectum D2cc: 72 Gy (57–82 Gy) Bladder D2cc: 74 Gy (56–92.8 Gy)
Shimbo 2023 (Oncol Lett; KORTUC + ISBT) [21]	11	Pelvic/vaginal recurrences	ISBT + KORTUC	EBRT: Not Mentioned; ISBT: 21–45.5 Gy in 3–7 fx; EQD2 54–75 Gy (calc)	CT/ultrasound-based	2-year LC: 79%	Not Mentioned	Not Mentioned	Not Mentioned	Not Mentioned
Ren 2022 (Front Onco) [22]	23	Local recurrence	CT-guided HDR ISBT + EBRT	EBRT: 30.0–50.4 Gy ISBT: 6–7 Gy × 4–7 fractions EQD2 for re-irradiation 64.0 Gy (range: 31.3–95.1 Gy) median cumulative EQD2 (for primary treatment and reirradiation) was 152.4 Gy (range: 97.8–200.9 Gy)	CT-guided	Not mentioned	1, 2, 3, 4-year PRS: 65.2%, 43.5%, 33.8%, and 27.1% respectively	G3–G4 toxicities:39.1%; Grade ≥3 rectal toxicity: 13.0%; Grade ≥3 urinary toxicity: 21.7%;	Tumor volume, tumor invasion organ number	median cumulative EQD2 D2cc of rectum and bladder was 115.0 Gy (range = 84.4–189.3 Gy) and 130.5 Gy (range = 95.5–173.5 Gy)

Abbreviations: HDR: high dose rate; ISBT: interstitial brachytherapy; EBRT: external beam radiotherapy; LPFS: local progression-free survival; 3D-PNCT: 3-dimensional printing noncoplanar template; OS: overall survival; GI: gastrointestinal; GU: genitourinary; PRS: post-relapse survival; *: EQD2 of OAR stands for the salvage treatment dose delivery only, because initial treatment involving 2D brachytherapy and radiation of OAR were incalculable.

## Data Availability

The data presented in this study are available in this article.
